# Integrating Cellular Immune Biomarkers with Machine Learning to Identify Potential Correlates of Protection for a *Trypanosoma cruzi* Vaccine

**DOI:** 10.3390/vaccines13090915

**Published:** 2025-08-28

**Authors:** Juan Cruz Gamba, Eliana Borgna, Estefanía Prochetto, Ana Rosa Pérez, Alexander Batista-Duharte, Iván Marcipar, Matías Gerard, Gabriel Cabrera

**Affiliations:** 1 Facultad de Bioquímica y Ciencias Biológicas, Universidad Nacional del Litoral, Santa Fe 3000, Argentina; gambajuancruz@gmail.com (J.C.G.); imarcipr@gmail.com (I.M.); 2 Research Institute for Signals, Systems and Computational Intelligence (CONICET-UNL), Facultad de Ingeniería y Ciencias Hídricas, Santa Fe 3000, Argentina; mgerard@sinc.unl.edu.ar; 3 Facultad de Ciencias Médicas, Universidad Nacional del Litoral, Santa Fe 3000, Argentina; 4 Instituto de Inmunología Clínica y Experimental de Rosario (IDICER-CONICET), Facultad de Ciencias Médicas, Universidad Nacional de Rosario, Santa Fe 3000, Argentina; 5 Centro de Investigación y Producción de Reactivos Biológicos (CIPReB), Facultad de Ciencias Médicas, Universidad Nacional de Rosario, Santa Fe 3000, Argentina; 6 Immunology and Allergy Group (GC01), Maimonides Biomedical Research Institute of Cordoba (IMIBIC), Reina Sofia University Hospital, University of Cordoba, Av. Menendez Pidal s/n, 14004 Cordoba, Spain

**Keywords:** chagas disease, machine learning, vaccine, correlate of protection, myeloid-derived suppressor cells, *Trypanosoma cruzi*, decision tree

## Abstract

**Background:** Chagas disease, caused by the protozoan parasite *Trypanosoma cruzi* (*T. cruzi*), remains a major public health concern in Latin America. No licensed vaccine exists to prevent or treat *T. cruzi* infection. Identifying correlates of protection (CoPs) could provide substitute endpoints to guide and accelerate vaccine development. Although most CoPs established to date are antibody-based, their utility has not been demonstrated in *T. cruzi* vaccine reports. Thus, this study aimed to explore alternative strategies considering the use of immune cells as potential CoPs. **Methods:** Mice were immunized with a vaccine candidate based on the *T. cruzi* trans-sialidase protein (TSf) and potentiated with 5-fluorouracil (5FU) to deplete myeloid-derived suppressor cells (MDSCs). Percentages of CD4^+^, CD8^+^, and CD11b^+^Gr-1^+^ cellular biomarkers were assessed by flow cytometry from the peripheral blood of immunized mice, which were subsequently challenged with a high dose of *T. cruzi.* A machine-learning (ML) model based on decision trees was applied to identify potential CoPs to predict survival by day 25 post-infection. **Results:** Individual biomarkers obtained from flow cytometry did not show strong predictive performance. In contrast, biomarker engineering led to a combination that integrated biomarkers rationally: summing the percentages of CD8^+^ and CD4^+^ cells and subtracting the percentage of CD11b^+^Gr-1^+^ MDSC-like cells (REB), enhanced the predictive capacity. Subsequent computational analysis and ML application led to the identification of a better and even improved potential Integrative CoP: $2\;*\;\%CD8^{+}+\;{\%CD4}^{+}\;-\;{\%CD11b}^{+}{\;Gr1}^{+}$(pICoP), which significantly improved the performance of a simple one-level decision-tree model, achieving an average accuracy of 0.86 and an average AUC-ROC of 0.87 for predicting survival in immunized and infected mice. **Conclusions**: Results presented herein provide evidence that integrating cellular immune biomarkers through rational biomarker engineering, together with ML analysis, could lead to the identification of potential CoPs for a *T. cruzi* vaccine.

## 1. Introduction

Chagas disease, caused by the protozoan parasite *Trypanosoma cruzi* (*T. cruzi*), is a significant public health issue, particularly in Latin America, where an estimated seven million individuals are infected, and 65–100 million people worldwide are at risk of infection [1,2]. The globalization of this disease has increased due to international migration, leading to cases in non-endemic regions [3]. Although therapeutic options such as benznidazole and nifurtimox are available for the acute phase, adverse effects are reported in up to 40% of treated patients, and their effectiveness in chronic Chagas disease is limited [4]. These limitations underscore the critical need for alternative approaches, including prophylactic and therapeutic vaccines.

Despite significant progress in vaccinology over the past decades [5,6], no licensed vaccine exists to prevent or treat *T. cruzi* infection [2,7]. Developing vaccines for this pathogen is particularly challenging due to its complex life cycle, substantial genetic variability [8,9], and its ability to evade and manipulate host immune responses [5,10,11,12,13]. Notably, *T. cruzi* employs multiple mechanisms to subvert the immune system during the acute phase of infection, thereby compromising the host’s ability to mount effective immune responses [14,15]. These factors, common to many pathogens lacking licensed vaccines, present obstacles that delay vaccine development [16,17].

In addition, assessing prophylactic or therapeutic vaccines that prevent *T. cruzi*-induced pathology is challenging due to the disease’s prolonged and variable progression. Approximately 30% of infected individuals develop Chagas cardiomyopathy over decades, complicating trial design and requiring long-term follow-up to assess endpoints such as disease progression and severe complications [7]. This extended timeline not only increases costs but also presents logistical and ethical challenges.

A valuable strategy to overcome these hurdles involves establishing correlates of protection (CoPs). This work adopts the definition proposed by Plotkin et al., 2012 [18], which states that a CoP is a measurable attribute statistically associated with protection [18]. CoPs can be further categorized as mechanistic (mCoPs), which directly mediate protection, and non-mechanistic (nCoPs), which serve as statistical substitutes [18]. In scenarios where measuring clinical endpoints is resource-limited or ethically constrained, CoPs could serve as substitute endpoints, facilitating the evaluation and advancement of vaccine candidates [19].

Interestingly, the identification of CoPs has relied almost exclusively on univariate analyses of antibody responses [20]. This traditional approach assumes that the sample size is sufficient to represent the entire population and typically employs regression methods to search for straightforward linear relationships between measured antibody levels and protection. However, despite extensive assessments of antibody levels in vaccine studies for *T. cruzi*, antibody-based CoPs have not been reported [9]. This suggests that new strategies may be necessary to guide the identification of potential CoPs for advancing *T. cruzi* vaccine development. To this end, broader approaches that consider not only antibody levels but also cellular immune responses may be required.

Machine learning (ML) is a set of techniques that enable computer systems to learn patterns from data without being explicitly programmed [21]. When a supervised learning strategy is employed, the performance of the fitted model can be evaluated using unseen data, allowing the predictive capability of the model to be estimated [22]. Additionally, the use of different partition strategies allows for correctly training models even with moderate sample sizes. These characteristics make ML models suitable for providing solutions to a wide range of problems in different disciplines [23,24,25]. Particularly, in the context of immunology, applying ML to immunological data holds transformative potential for vaccine development, with the identification of CoPs being one of its promising applications [24].

Machine learning encompasses a wide range of algorithms based on different principles. Among them, decision trees are a family of methods based on recursive binary partitioning of the feature space that creates a hierarchical structure of decision rules organized in nodes—with each internal node representing a decision based on a feature value and each leaf node containing the final prediction—that split the data into increasingly homogeneous subsets until reaching a classification or regression output [26,27]. This family has gained increasing importance in biomedical research due to their interpretability, ability to handle both numerical and categorical variables without preprocessing, robustness to outliers, and capacity to automatically approximate complex patterns and capture feature interactions [27]. This means that in cases where class separation is reflected in the data structure, even low-complexity trees involving a few branches can identify separation criteria.

Previously, we developed a trans-sialidase (TS)-based vaccine formulated with a cage-like particle adjuvant (ISPA), which elicited a potent humoral and cellular immune response associated with robust protection [28,29]. Given the extensive evidence describing *T. cruzi*’s ability to markedly affect the regulatory immune system, along with findings indicating that vaccines may influence not only the effector arm of the immune system but also its regulatory counterpart, our group incorporated the study of the regulatory immune response as a novel approach to enhance a vaccine against *T. cruzi*. In this context, we reported that our vaccine candidate, in addition to eliciting a potent effector immune response, also induced a parallel increase in myeloid-derived suppressor cells CD11b^+^ Gr-1^+^ (MDSCs) in the spleen, which could limit, at least in part, vaccine efficacy [28,30]. Furthermore, to enhance the TSf-ISPA vaccine, protocols using 5-fluorouracil (5FU) to deplete MDSCs were developed. The vaccine formulation that incorporated two doses of 5FU (double 5FU TSf-ISPA) overcome the other formulations based on one or none 5FU doses, achieving the highest protection against high lethal *T. cruzi* challenges, and conferring protection across different infection routes, against parasite strains from different discrete typing units, and in mouse models with different genetic background such as BALB/c and C57BL/6 mice [30,31]. Interestingly, the increase in the protective capacity of the double 5FU TSf-ISPA vaccine significantly decreased MDSCs, increased CD4 and CD8 responses in the spleen and lymph nodes as compared to the other vaccine formulations, but did not significantly affect antibody levels [31].

Considering that identifying CoPs against the complex pathogen *T. cruzi* may require novel approaches and that the double 5FU TSf-ISPA vaccine achieved the highest protective capacity by targeting a suppressive cell population, the general objective of this work was therefore to apply ML to identify potential CoPs for survival conferred by this vaccine candidate, not by prioritizing antibody levels but by assessing and integrating cellular biomarkers such as CD8^+^, CD4^+^, and CD11b^+^ Gr-1^+^ MDSC-like cells from peripheral blood.

## 2. Materials and Methods

### 2.1. Mice and Parasites

Female BALB/c mice aged 6–8 weeks were used in all experimental procedures. The animals were sourced from the Centro de Medicina Comparada (CMC) at the Universidad Nacional del Litoral (UNL), Argentina. All animal experiments were conducted in accordance with protocols approved by the Advisory Committee of Ethics and Research Safety (CAESI) of the Facultad de Bioquímica y Ciencias Biológicas, UNL, adhering to international ethical standards for biomedical research involving animals (CAESI 2022-1/22).

Tulahuen strain blood trypomastigotes were harvested from the blood of Cb1-infected mice, as previously described [28,30,31], under protocols approved by the Institutional Committees on Bioethics and Biosecurity (Res. Nº: 0805/2020 and 2142/2024).

### 2.2. Treatments and Immunization Schedules

Mice were divided into three groups, each assigned a different treatment scheme.

The experimental group of double 5FU TSf-ISPA mice was immunized with three subcutaneous doses, administered biweekly, each containing 10 μg of a trans-sialidase antigen (TSf) (GenBank AJ276679.1), prepared as previously described [28], along with 2.5 μL of ISPA as an adjuvant (group TSf-ISPA). Additionally, these mice received intraperitoneal (i.p.) injections of 5-fluorouracil (5FU) both prior to each TSf-ISPA immunization and again seven days afterward.

The TSf-ISPA control group received the same immunization protocol, except that phosphate-buffered saline (PBS) was administered instead of 5FU. The PBS-control group received equal volumes of PBS in place of both TSf-ISPA and 5FU. According to the high *T. cruzi* dose used, PBS-treated control mice showed 100% mortality, and they were not included in ML analysis. TSf-ISPA-treated control mice showed high mortality (94%) and imbalanced data regarding survival; they were used as a control for MDSC increases in the absence of 5FU-treatment, as previously described [30,31].

ISPA is a cage-like particle adjuvant formulated as previously described, using dipalmitoylphosphatidylcholine (DPPC), cholesterol (CHOL), stearylamine (STEA), tocopherol (TOCOP), and Quillaja saponaria extract (QuilA) [29]. 5FU (Sigma-Aldrich, St. Louis, MO, USA) was administered at a dose of 50–60 mg/kg, a regimen previously shown to selectively deplete myeloid-derived suppressor cells (MDSCs) in several murine studies [32,33], including during *T. cruzi* infection [34,35].

Three independent experiments were performed. Experiment one: Double 5FU TSf-ISPA, n = 8; TSf-ISPA, n = 9; PBS n = 5. Experiment two: Double 5FU TSf-ISPA, n = 15; TSf-ISPA, n = 14; PBS n = 4. Experiment three: Double 5FU TSf-ISPA, n = 8; TSf-ISPA, n = 8; PBS n = 2. Total n: double 5FU TSf-ISPA = 31; TSf-ISPA n = 31, PBS n = 11.

We have previously reported that administration of TSf or ISPA alone does not confer protective capacity [28].

### 2.3. Infection Protocol, Parasitemia, and Survival

BALB/c mice were challenged i.p. with 1600 to 1700 bloodstream trypomastigotes of the Tulahuen strain, 15 days after the last TSf-ISPA immunization. Parasitemia was monitored on day 15 post-infection (p.i.) as a control of vaccine efficacy and as a potential target variable in future analysis. Survival was recorded until day 35 p.i.

### 2.4. Flow Cytometry

The protocol was performed as previously described [36]. Briefly, mice were punctured in the facial vein using a sterile lancet, and three drops of blood were collected into an Eppendorf tube containing 10 μL of sodium heparin. Fifty microliters of peripheral blood were transferred to a 15 mL Falcon tube and lysed with 2 mL of sterile distilled water for 30 s. The volume was then adjusted to 8 mL with physiological saline solution (PSS) containing 10% fetal bovine serum (FBS, Internegocios S.A, Mercedes, Argentina), followed by centrifugation at 300× *g* for 5 min at room temperature. The supernatant was carefully discarded, and the cell pellet was resuspended in 120 μL of PSS with 3% FBS and 0.1% sodium azide.

Between 0.75 and 1 × 10^5^ cells were incubated with 0.1 μg of anti-FcγR III/II blocking antibody (BD-Biosciences, San Diego, CA, USA) in 100 μL of the same buffer for 10 min at 4 °C in the dark. Cells were then stained with 0.2 μg of fluorochrome-conjugated antibodies—anti-CD8, anti-CD11b, anti-Gr-1, and anti-CD4 (BD-Biosciences, San Diego, CA, USA)—in appropriate combinations of fluorochromes. After 40 min at 4 °C, cells were washed with 1 mL of PSS with 3% FBS and 0.1% azide and centrifuged again at 300× *g* for 5 min.

Samples were acquired on a multicolor flow cytometer, Accuri C6 Plus (BD-Biosciences), and analyzed using FlowJo v10.0.7 Software.

### 2.5. Leukocyte Trafficking Assay: Adoptive Transfer and Flow Cytometry Analysis

Peripheral blood cells were isolated from BALB/c mice previously immunized with the experimental vaccine TSf-ISPA. Following collection, red blood cells were lysed using a similar protocol as described for flow cytometry [36]. Leukocytes were resuspended in PSS containing 0.5% FBS.

A total of 8 × 10^6^ cells were labeled with CFSE (5 µM; ThermoFisher, Waltham, MA, USA) by incubation at 37 °C for 20 min in the dark. Labeling was stopped by adding PSS supplemented with 10% FBS, followed by two centrifugations at 300× *g* with a 5-min incubation at 37 °C between them. The efficiency of CFSE labeling was verified by flow cytometry. Labeled cells were then adoptively transferred into recipient BALB/c mice previously immunized with TSf-ISPA. After 24 h, adoptively transferred and control mice were sacrificed, and spleen cells were prepared for flow cytometry. After Fc receptor blocking, cells were stained with fluorochrome-conjugated monoclonal antibodies against CD4 (APC), CD8 (PerCP-Cy5.5), or against CD11b (PerCP-Cy5.5) in separate analyses. Flow cytometric data were acquired and analyzed as previously described for flow cytometry.

### 2.6. Dataset Construction

The dataset was built using the percentages of CD4^+^ T cells, CD8^+^ T cells, and CD11b^+^ Gr-1^+^ myeloid-derived suppressor cell (MDSC)-like populations, obtained from flow cytometry analysis of peripheral blood samples collected from BALB/c mice immunized with the double 5FU TSf-ISPA vaccine, together with survival outcomes following *T. cruzi* infection. These data correspond to three independent experiments.

Each entry in the dataset represented an individual mouse and included the immunological features—i.e., the immunological biomarkers measured by flow cytometry—along with a binary variable indicating whether the mouse survived or died after infection. Only mice with complete flow cytometry data and defined survival outcomes were included. The dataset was subsequently used to train a machine-learning model aimed at identifying the most informative biomarker as a potential CoP.

### 2.7. Machine-Learning Model

In supervised machine learning, data must be split into training and testing subsets to enable the algorithm to learn from known outcomes and subsequently evaluate its predictive performance. Decision trees are supervised machine-learning algorithms that construct hierarchical models through recursive data partitioning for classification and regression tasks. The structure comprises three key components: internal nodes representing feature-based decision points, branches indicating decision outcomes, and leaf nodes containing final predictions. Starting from a root node encompassing the entire dataset, the algorithm recursively splits data into homogeneous subsets using splitting criteria such as Gini impurity or information gain. At each internal node, the optimal feature and threshold are selected to maximize class purity. This partitioning continues until stopping criteria are met, such as perfect node purity or maximum depth. Predictions are made by traversing from root to leaf following decision paths determined by input feature values. The general structure and functioning of decision trees have been extensively described in the literature, which provides the theoretical foundations for their application in supervised learning. Comprehensive discussions on decision-tree learning and implementation can be found in the specialized literature [26,27].

In this work, a tree with depth 1, known as a decision stump, was used to classify the outcome of vaccinated mice treated with 5-FU following *T. cruzi* challenge, specifically distinguishing between survival and death. The input features consisted of immunological biomarkers—%*CD*4^+^, %*CD*8^+^, and %*CD*11*b*^+^ *Gr*1^+^—which were evaluated as potential CoP. In this classification task, one binary split was performed by a single root node based on an optimal biomarker threshold, and exactly two leaf nodes with class predictions were generated. The decision boundary was defined by a biomarker value threshold that created a linear separation in the biomarker space. The classification mechanism was based on comparing input biomarker values against this predetermined threshold, with observations being assigned to different classes based on whether the split point was exceeded. Hierarchical complexity was eliminated by this unidimensional decision rule. Scikit-learn 1.5.2 and Python 3.12.1 were used to implement this algorithm.

Since supervised machine learning relies on learning patterns from labeled data, the model was trained using a portion of the available samples and subsequently tested on unseen data to evaluate its generalization capacity. To this end, a stratified five-fold cross-validation procedure was employed. This approach ensured that both outcome classes—survival and death—were proportionally represented in each fold. In every iteration, one fold was reserved for testing while the remaining four folds were used for training. This procedure was repeated five times, such that each observation served once as a test instance and four times in training. Performance metrics were averaged across folds to yield a robust estimate of model accuracy, mitigating the influence of any single data partition and reducing the risk of overfitting.

Supplementary Material SI describes the data used to build the dataset employed for training and testing the decision-tree model. The source code of the computational experiments, including the implementation of the decision stump model and the procedures for training, testing, and performance evaluation, is available along with documentation in the associated GitHub repository.

### 2.8. Performance Evaluation Metrics

Results are reported and analyzed using standard binary classification performance metrics [37]. Each pattern represents the biomarker vector associated with an individual mouse specimen and its corresponding survival outcome. True positive (TP) instances occur when both predicted and actual classifications indicate survival. True negative (TN) instances correspond to correctly predicted non-survival cases where the actual outcome is death. False positive (FP) instances represent misclassifications where survival is predicted, but the actual outcome is death. False negative (FN) instances occur when non-survival is predicted, but the mouse survives. These confusion matrix elements enable a comprehensive evaluation of classifier performance through derived metrics, including accuracy, precision, recall, and F1-score.

Based on these definitions, three metrics were used to analyze classifier performance. Accuracy measures the proportion of correctly classified instances across all predictions, calculated as Accuracy=(TP+TN)/(TP+TN+FP+FN). This metric provides an overall assessment of classification correctness, but can be misleading in imbalanced datasets where one class significantly outnumbers the other. F1-score represents the harmonic mean of precision and recall, computed as F1=2 ∗ (Precision ∗ Recall)/(Precision+Recall), where Precision=TP/(TP+FP) and Recall=TP/(TP+FN). This metric balances precision and recall, providing robust performance evaluation, particularly valuable for imbalanced classification problems. AUC-ROC quantifies the area under the receiver operating characteristic curve, which plots true positive rate against false positive rate across various classification thresholds. AUC values range from 0 to 1, where 0.5 indicates random performance and 1.0 represents perfect classification capability regardless of class distribution.

These performance metrics provide a comprehensive evaluation framework for assessing the decision-tree classifier’s ability to identify potential CoP in vaccine studies against *T. cruzi* infection. Accuracy offers overall classification performance, F1-score ensures balanced evaluation of survival prediction capability, and AUC-ROC demonstrates the model’s discriminative power across different decision thresholds, collectively validating the reliability of identified biomarker combinations as potential CoP.

### 2.9. Simulation of Input Variability to Evaluate the Potential CoP Decision Logic

In order to assess whether the classification logic defined by a potential integrative correlate of protection (pICoP) remains effective when applied to input data differing from the original measurements, an analysis based on simulated input variability was performed. The original flow cytometry-derived measurements—%*CD*4^+^, %*CD*8^+^, and %*CD*11*b*^+^ *Gr*-1^+^ MDSC-like cells—were systematically modified by introducing random stochastic variation within three predefined ranges: ±0–5%, ±5–10%, and ±10–15% of their original values. For each level of variability, a new dataset was generated by applying independent noise to each variable across all samples. The potential CoP was recalculated using the same linear combination, and a new decision-tree model was trained and tested on each modified dataset, following the same procedures used in the original model training and evaluation. This process was repeated 100 times per variability condition.

The area under the ROC curve (AUC-ROC) was used as the primary performance metric. To quantify the impact of variability, the number of iterations in which the AUC-ROC exceeded the threshold of 0.7 [38]—a value accepted as indicative of acceptable discriminatory performance in biomedical classification tasks—was recorded for each level of input variation.

### 2.10. Statistical Analysis

For comparisons between two independent groups, the Mann–Whitney U test (also known as the Wilcoxon rank-sum test) was used. This non-parametric method is suitable for data that do not follow a Gaussian distribution, as it does not require the assumption of normality. Instead of comparing means, the Mann–Whitney U test assesses whether one distribution tends to yield systematically higher (or lower) values than the other. All observations from both groups are ranked jointly, and the U statistic is calculated. This statistic quantifies how often values from one group precede those from the other in the ranked data, providing a clear measure of the relative position between the two distributions. Depending on prior knowledge regarding the expected direction of change, either one- or two-tailed tests were applied.

For survival analyses, the Mantel–Cox (log-rank) test was employed. This non-parametric approach assesses differences in survival curves by comparing the observed and expected number of events at each time point under the null hypothesis of equal survival functions. It assumes proportional hazards and is widely used in both clinical and preclinical time-to-event analyses.

These statistical analyses were performed using GraphPad Prism 8.0.1 (GraphPad Software, San Diego, CA, USA). Statistical significance was denoted as follows: (*) for *p* < 0.05, (**) for *p* < 0.01, and (***) for *p* < 0.001.

Since the performance metrics were derived from multiple iterations of the model using resampled data, independence between observations could not be assumed. Consequently, permutation-based approaches were selected, as they provide valid statistical inference under dependency structures and do not rely on parametric distributional assumptions.

A permutation test against randomness was carried out in order to assess whether a detected pattern could have arisen by chance. The test statistic—such as AUC-ROC—was computed on the original data. Next, sample labels or values were randomly permuted a large number of times to break any real association, thereby generating an empirical null distribution of the test statistic. The *p*-value was derived as the proportion of permutations yielding a statistic as extreme or more extreme than the observed one. As a fully non-parametric method, this approach ensures robustness against non-Gaussian data.

To compare performance metrics (e.g., accuracy, AUC-ROC, F1-score) between two biomarkers, a two-group mean-difference permutation test was performed. Group labels were shuffled, and for each permutation, the difference in means was recalculated, forming a null distribution of mean differences. The empirical *p*-value was calculated as the fraction of permuted differences whose absolute value was at least as large as the observed difference. This method effectively tests the null hypothesis wherein the mean differences are solely due to chance.

To quantify the uncertainty surrounding the estimated performance metric, confidence intervals (CIs) were computed using the bootstrap method. This non-parametric approach involves resampling the dataset with replacement a large number of times, recalculating the statistic of interest for each resample. The resulting empirical distribution was then used to derive percentile-based confidence intervals—for example, the 2.5th and 97.5th percentiles for a 95% CI. Given its minimal reliance on distributional assumptions, the bootstrap method is particularly appropriate in the context of model evaluation.

All permutation-based statistical analyses and confidence interval estimation were implemented in Python. The source code used to perform these tests is available in the project’s GitHub repository. Statistical significance was denoted as follows: (*) for *p* < 0.05.

## 3. Results

### 3.1. Experimental Assays for Dataset Construction

**Figure 1:**
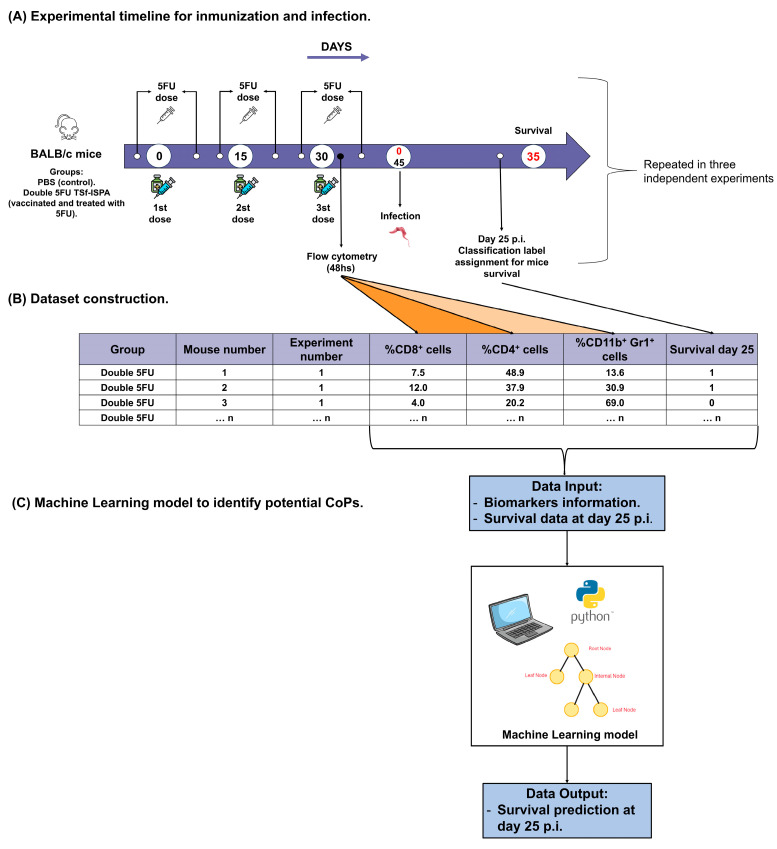
Workflow including experimental timeline, dataset construction, and machine-learning analysis. The study can be divided into three steps: (**A**) Experimental timeline for immunization and infection, for data collection: Female BALB/c mice were assigned to control (PBS) or vaccinated (double 5FU TSf-ISPA) groups. The vaccinated group received three subcutaneous doses of TSf-ISPA (on days 0, 15, and 30), each preceded and followed by intraperitoneal 5FU administration to reduce MDSC expansion. Flow cytometry of peripheral blood was performed 48 h after the final TSf-ISPA dose. All groups were intraperitoneally infected with 1600–1700 Tulahuen strain trypomastigotes. Survival was recorded until day 35 p.i.. The experiment was repeated three times to collect a useful number of animals. (**B**) Dataset construction: Flow cytometry data and corresponding survival outcomes for each mouse on day 25 p.i. were used to construct a dataset including the three assays performed. (**C**) The dataset constructed was used to train and test a decision-tree classification model to identify potential CoPs associated with survival.

To obtain the data necessary for applying ML to identify potential CoPs, three independent experiments were conducted, each including the following tasks: immunization with double 5FU TSf-ISPA, flow cytometric assessment of potential CoPs in peripheral blood, *T. cruzi* infection, and survival analysis (Figure 1A). A dataset was completed with information on each mouse (Figure 1B), including the percentages of CD4^+^, CD8^+^, and CD11b^+^ Gr-1^+^ cells in peripheral blood 48 h after the last immunization dose, as well as survival following a challenge with a high dose of Tulahuen *T. cruzi* parasites (Supplementary Material SI). This dataset was used to construct a machine-learning model (Figure 1C).

Previously, we reported that the double 5FU TSf-ISPA formulation increased the number of CD4^+^ and CD8^+^ cells in the spleens of vaccinated mice. Additionally, CD11b^+^Gr-1^+^ splenocytes increased by day 7 post-immunization (p.i.) but were subsequently depleted through the double 5FU treatment [30,31]. Since the analysis of CoPs requires measuring immune cell biomarkers without sacrificing the animals, a protocol was developed to assess these populations 48 h p.i. in peripheral blood instead of in the spleen, allowing subsequent challenge with *T. cruzi* [36].

**Figure 2:**
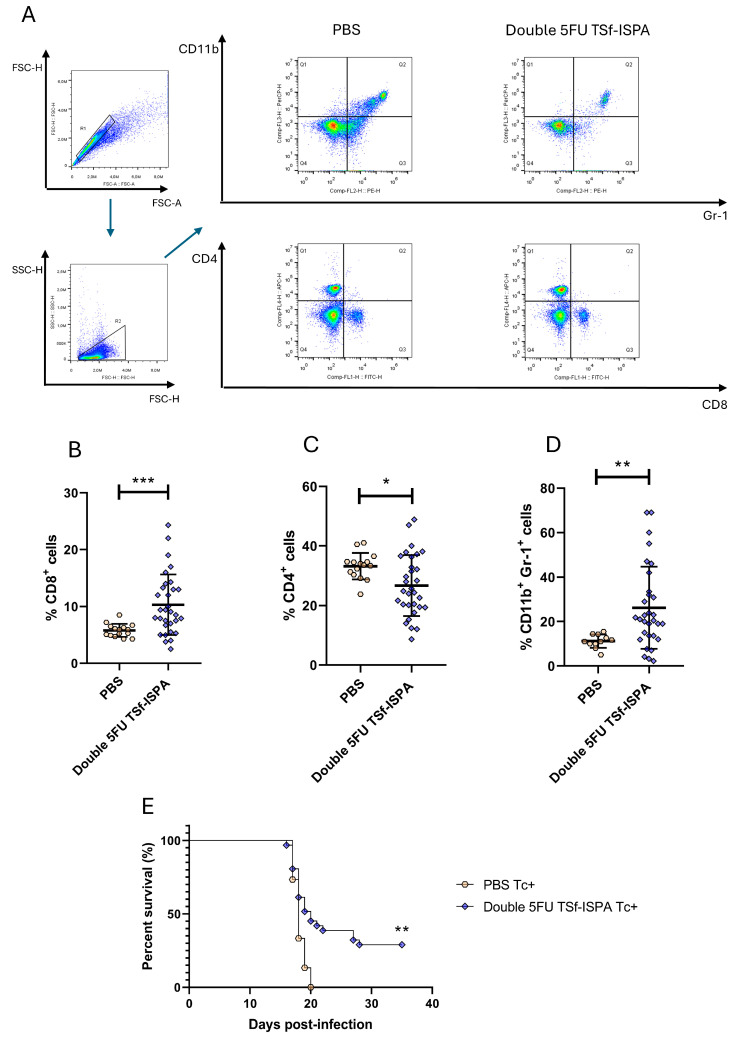
Evaluation of peripheral blood immune cell populations during the immunization protocol and survival of mice following *T. cruzi* infection. Female BALB/c mice were immunized with double 5FU TSf-ISPA or received PBS as a control. Peripheral blood was collected 48 h after the last immunization dose and analyzed by flow cytometry. (**A**) Representative dot plots showing the gating strategy used to identify CD4^+^, CD8^+^, and CD11b^+^Gr-1^+^ cells in mice treated with PBS or double 5FU TSf-ISPA. (**B**–**D**) Percentages of CD4^+^ (**B**), CD8^+^ (**C**), and CD11b^+^Gr-1^+^ (**D**) cells in 50 μL of peripheral blood from PBS or double 5FU TSf-ISPA-treated mice. Data are expressed as means ± standard deviations. (**E**) Survival curve until day 35 p.i.. Results include data from three independent experiments. Mann–Whitney test was used for (**B**–**D**), and Mantel–Cox log-rank test for (**E**). * *p* < 0.05, ** *p* < 0.01, *** *p* < 0.001.

Figure 2A depicts representative dot plots of CD4^+^, CD8^+^, and CD11b^+^ Gr-1^+^ MDSC-like cells in experimental Double 5FU TSf-ISPA and PBS-treated control mice. Figure 2B, C, and D describe the alterations caused in the experimental group of double 5FU TSf-ISPA mice regarding the percentage of CD4^+^, CD8^+^, and CD11b^+^ Gr-1^+^ cells measured in 50 μL of peripheral blood. As shown in Figure 2B, the mean percentage of CD4^+^ cells of vaccinated mice is decreased in peripheral blood as compared to PBS-treated mice. In contrast, the mean percentage of CD8^+^ cells is significantly increased in vaccinated mice as compared to controls (Figure 2C). Finally, Figure 2D illustrates that the mean percentage of CD11b^+^ Gr-1^+^ MDSC-like cells is significantly increased in vaccinated mice as compared to PBS-treated mice [28,30,31]. We have previously reported that double 5FU treatment is required to deplete CD11b^+^ Gr-1^+^ MDSCs in the spleen of TSf-ISPA vaccinated mice [31]. The capacity of 5FU to target CD11b^+^ Gr-1+ MDSC-like cells also in peripheral blood was controlled using control mice vaccinated only with TSf-ISPA, and not treated with 5FU. As shown in Supplementary Figure S1, CD11b+ Gr-1+ MDSC-like cells increased significantly in TSf-ISPA vaccinated mice, whereas double 5FU TSf-ISPA treatment significantly decreased the levels of CD11b+ Gr-1+ MDSC-like cells, supporting the functionality of 5FU to target these cells in peripheral blood.

Taken together, these results suggest that double 5FU TSf-ISPA treatment significantly affects the levels of CD4^+^, CD8^+^, and CD11b^+^ Gr-1^+^ MDSC-like cells in peripheral blood as compared to PBS treatment, and varies for each particular individual in a wide range of values.

Given that the suppressive capacity of splenocyte MDSCs has already been established in our model [31], a migratory assay was performed to support that CD11b^+^Gr-1^+^ MDSC-like cells from peripheral blood comprise cells capable of homing to the spleen, as has been described in other studies [39,40]. For this purpose, peripheral blood was obtained from immunized mice 48 h after the last dose, CFSE-labeled, and then adoptively transferred into different immunized recipient mice. As shown in Supplementary Figure S2, CFSE^+^ CD11b^+^ Gr-1^+^ cells were recovered from the spleen of recipient mice, providing evidence that the analyzed peripheral blood populations contain cells capable of homing to the spleen, where their suppressor function has already been shown [31].

Finally, double 5FU TSf-ISPA–immunized mice were challenged with *T. cruzi* to assess survival in the same animals from which immune cell data had been collected by flow cytometry. Figure 2E shows that double 5FU TSf-ISPA mice survived significantly longer against a lethal dose of *T. cruzi* compared to PBS-treated mice. The considerable variation in individual percentages of CD4^+^, CD8^+^, and CD11b^+^ Gr-1^+^ MDSC-like cells, along with variable survival rates, raised the possibility of identifying one or more specific biomarkers that could serve as a CoP with the capacity to predict survival outcomes in immunized and infected mice.

#### Criteria Definition for Mice Survival

To develop ML models for searching potential CoPs, it is usually beneficial to have balanced groups of mice regarding the target variable that is desirable to predict, in this case, survival. A dose of parasites that causes 100% survival of vaccinated mice would not be useful for predicting CoPs, as every biomarker would eventually correlate with survival. Similarly, a very high dose that causes 100% mortality would not be useful, as no biomarker would correlate with survival. The selected dose of 1600–1700 parasites resulted in a final survival rate of 29% in vaccinated mice by day 35 post-infection (p.i.). However, final survival does not account for the timing of death, which may offer more biologically and statistically informative insights related to protection. Time-to-event data are better captured by statistical tests that consider the area under the survival curve, reflecting the overall duration of survival. To incorporate this temporal information, a classification criterion was developed: mice vaccinated with double 5FU TSf-ISPA were assigned a value of “0” if they died before day 25 p.i., and “1” if they survived beyond day 25 p.i. This classification criterion was selected, as it not only incorporates survival time but also resulted in a more balanced distribution (60% vs. 40%), making it more appropriate for training and evaluating machine-learning models.

### 3.2. Exploratory Analysis

An exploratory analysis was conducted to assess whether there exist differences between double 5FU TSf-ISPA mice levels of CD4^+^, CD8^+^, or CD11b^+^ Gr-1^+^ MDSC-like cells when comparing survival before and after day 25 p.i. (survival criterion).

**Figure 3:**
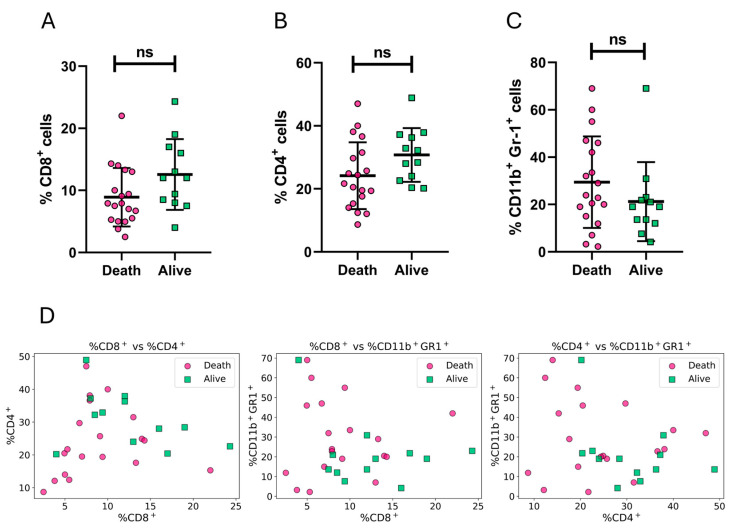
Exploratory analysis of peripheral blood immune cell populations in relation to survival outcome after *T. cruzi* infection. Double 5FU mice were assigned the label corresponding to their survival status on day 25 post-infection. (**A**–**C**) Percentages of CD8^+^ T cells (**A**), CD4^+^ T cells (**B**), and CD11b^+^Gr-1^+^ myeloid-derived suppressor-like cells (MDSC-like) (**C**), in peripheral blood 48 h after the last immunization dose, from mice that died before (fuchsia circles) or survived beyond (green squares) day 25 p.i.. Although trends were observed, none of these differences reached statistical significance (ns; Mann–Whitney test). (**D**). Pairwise scatter plots showing relationships between two immune parameters: %*CD*8^+^ vs. %*CD*4^+^ (left), %*CD*8^+^ vs. %*CD*11*b*^+^ *Gr*-1^+^ (middle), and %*CD*4^+^ vs. %*CD*11*b*^+^ *Gr*-1^+^ (right). Data are expressed as means ± standard deviations. Results include data from three independent experiments. Mann–Whitney test was used for (**A**–**C**).

Mice that survived showed trends supporting that a higher percentage of CD8^+^ and CD4^+^ cells in peripheral blood correlates with survival, whereas lower levels of CD11b^+^ Gr-1^+^ MDSC-like cells are associated with survival, although statistical significance was not reached in these analyses (Figure 3A–C). An exploratory analysis using pairwise scatter plots of the measured biomarkers did not generate a clear visual separation of the target variable in immunized and infected mice (Figure 3D).

The fact that no individual cellular biomarker seems capable of performing as a clear CoP of protection by itself was in line with the current lack of CoPs described in the literature to date.

### 3.3. Rational Biomarker Engineering

An approach to continue searching for potential CoPs involves generating new combinations of the original features through a process known as feature engineering. In our study, these features correspond to immunological biomarkers, as described in the Methods Section. This search can be conducted by a computational exploration or guided by a rational approach. Since our previous data provided support to the notion that immunization may stimulate not only the effector response but also suppressive cells with the potential to limit the efficacy of vaccination, in this work, we first followed the evidence and rationally designed a new engineered biomarker based on knowledge gained from prior experience.

Thus, on the basis of a comprehensive view of the immune system, the following broad assumptions were made: CD4^+^ and CD8^+^ cells from peripheral blood primarily would include cells that can form part of the effector response, whereas CD11b^+^ Gr-1^+^ MDSC-like cells in the context of immunization with our vaccine candidate mainly would consist of cells with suppressive potential, consistent with migratory capacity to the spleen and our previous findings on CD11b^+^ Gr-1^+^ MDSC splenocytes [28,30,31]. It was then assumed that subtracting the percentage of MDSC-like cells from the sum of the percentages of biomarkers associated with the effector response would better reflect the protective capacity of a vaccine compared to using these biomarkers individually, as follows:

(1) $${REB\;=\%CD8}^{+}+\;{\%CD4}^{+}\;-\;{\%CD11b}^{+}{\;Gr1}^{+},$$

where REB corresponds to Rational Engineered Biomarker, %*CD*8^+^ and %*CD*4^+^ correspond to the percentages of CD8^+^and CD4^+^ T cells, respectively, in peripheral blood. %*CD*11*b*^+^ *Gr*1^+^ represents the percentage of CD11b^+^ Gr-1^+^ MDSC-like cells also measured in peripheral blood.

**Figure 4:**
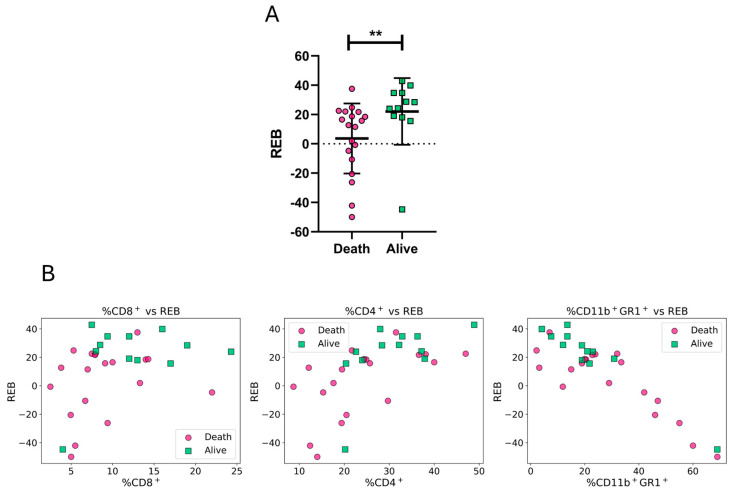
Rationally engineered biomarker based on effector-related and suppressor-related immune biomarkers. (**A**) Double 5FU mice were assigned the label corresponding to their survival status on day 25 post-infection. The engineered biomarker is calculated as the sum of CD8^+^ and CD4^+^ T cell percentages minus the percentage of CD11b^+^Gr-1^+^ MDSC-like cells in peripheral blood 48 h after the last immunization dose: ${\%CD8}^{+}+\;{\%CD4}^{+}\;-\;{\%CD11b}^{+}{\;Gr1}^{+}$(REB). Fuchsia circles represent individual mice that died before day 25, and green squares represent those that survived. (**B**) Pairwise scatter plots showing the relationship between the engineered biomarker and each of its constituent biomarkers. Each point corresponds to a single mouse, colored and shaped by survival outcome as in panel (**A**). Data are expressed as means ± standard deviations. Results include data from three independent experiments. Mann–Whitney test was used for (**A**). ** *p* < 0.01.

Figure 4A shows that this new engineered biomarker, developed on the basis of rational assumptions, significantly increased in immunized mice that survived day 25 p.i. as compared to mice that died before day 25 p.i. In consistent with this result, pairplots of this engineered biomarker with other individual biomarkers revealed clearer visual separations (Figure 4B).

### 3.4. Assessment of individual biomarkers and the rationally engineered biomarker (REB) as potential CoPs

In the next step, all individual biomarkers, along with the rational engineered biomarker (REB), were assessed as potential CoPs, using decision trees due to their previously described advantages. Accuracy and AUC-ROC metrics were used to assess the global performance of the models. In addition, considering that the data were moderately imbalanced (60% mortality vs. 40% survival), and the importance of predicting appropriately which mice would survive, special emphasis was placed on the model’s ability to correctly predict the minority class (i.e., surviving mice). To this end, the F1-score for mice that survived by day 25 p.i. was also calculated. This metric, which harmonizes precision and recall, is particularly informative in this context, as it reflects both the model’s capacity to identify true survivors (recall) and the reliability of those predictions (precision).

To provide a better estimation of model performance, 95% confidence intervals (CIs) were calculated for the average of each metric. These intervals were computed as described in the methods, and all reported means fall within their respective 95% confidence intervals. The complete calculation and corresponding results are available in the source code provided in the Supplementary Material. As shown in Table 1, the REB outperformed each original biomarker on average.

### 3.5. Systematic Search of Weighted Combinations for the Engineered Biomarker

Since this rational approach to identifying a potential CoP led to an improvement in model performance, a further refinement of the engineered biomarker was pursued. Previous findings from our group and others [30,31,34,41,42] have suggested that CD8^+^ T cell responses may play a critical role in protection against *T. cruzi*. Based on this knowledge, we hypothesized that, when combining immunological variables into a single composite biomarker, CD8^+^ might exert a comparatively greater influence. To explore the scenario where CD8^+^ dominates the combination and compare its discriminative performance against other possibilities, different weighting schemes for the three biomarkers were considered. All possible combinations of weights for %*CD*8^+^, %*CD*4^+^, and %*CD*11*b*^+^ *Gr*-1^+^ MDSC-like cells were analyzed, considering values in the range [–6, +6], with increments of 0.5. These weights were used to generate the following linear combination:

(2) pICoP =α  ∗ %CD8⁺+β ∗ %CD4⁺ + γ ∗ %CD11b⁺Gr1⁺,

where pICoP correspond to Potential Integrative Correlate of Protection, alpha (α), beta (β), and gamma (γ) are the coefficient weights for %*CD*8^+^, %*CD*4^+^ cells, and %*CD*11*b*^+^ *Gr*-1^+^, respectively. Each of the combinations built from Equation (2), corresponding to different engineered biomarkers, was evaluated using our stump decision-tree model.

The resulting engineered biomarkers were assessed based on both F1-score and AUC-ROC, and the combinations yielding the highest values for these metrics were selected for further analysis. This exhaustive search enabled the identification of combinations where CD8^+^ effectively emerges as a primary contributor, in alignment with its hypothesized functional relevance.

Regardless of the magnitude of the coefficients, most of the engineered variables that yielded the best performance metrics were based on the same type of combination that was used during rational design (shown in Supplementary Material SII). This supports the idea that the rational strategy (REB)—summing variables more associated with the effector response and subtracting that one related to the regulatory/suppressor response—may represent a valuable strategy for identifying potential CoPs in the context of vaccine research. Additionally, the best performing variables have a higher weight coefficient for CD8^+^ as compared to the other variables, supporting the relevance of the CD8^+^ response as being markedly important for vaccine efficacy.

Although more than one biomarker of the Supplementary Material SII demonstrated potentially good performances, the potential integrative CoP: ${2\;*\;\%CD8}^{+}+{\%CD4}^{+}-{\%CD11b}^{+}{Gr1}^{+}$ (pICoP) was selected for further analysis due to its strong performance, simplicity, and statistical significance, as assessed by a permutation test against randomness (*p* < 0.05). Additionally, all metrics significantly improved in the pICoP compared to the REB. Table 2 shows improvements in average performance metrics, with accuracy rising from 0.72 to 0.86, AUC-ROC from 0.70 to 0.87, and F1-score from 0.61 to 0.83. As before, 95% confidence intervals (CIs) were computed for the mean of each metric. A permutation test was performed for each metric to compare the two engineered biomarkers, revealing statistically significant differences in all cases (*p* < 0.05).

The final evaluation results of the pICoP training and test are presented in Supplementary Table S1.

**Figure 5:**
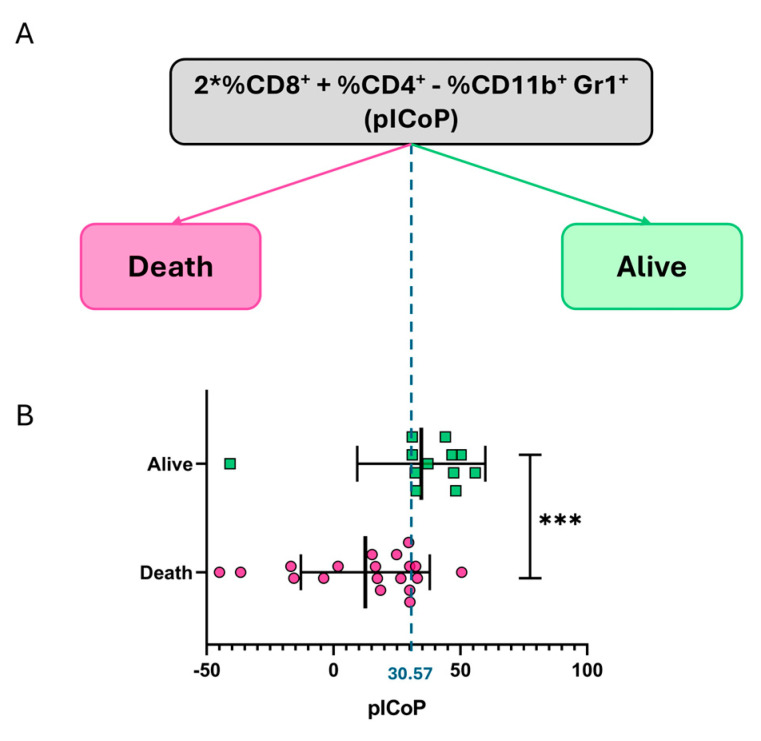
Example of a resulting classification tree trained on the engineered biomarker and its capacity to discriminate survival outcomes. (**A**) Representative decision tree (depth = 1) obtained from training on the final potential integrative CoP (pICoP), calculated as ${2\;*\;\%CD8}^{+}+\;{\%CD4}^{+}\;-\;{\%CD11b}^{+}{\;Gr1}^{+}$. The model establishes a linear decision boundary with an average threshold of 30.57 ± 0.31, effectively separating mice into two classes based on survival status by day 25 post-infection. This simplified tree exemplifies how the newly defined biomarker serves as a discriminator of protection. (**B**) Distribution of the pICoP in individual mice, stratified by survival outcome. Fuchsia circles represent animals that died before day 25, and green squares represent those that survived (Mann–Whitney U test, *** *p* < 0.001). Data are expressed as means ± standard deviations. Results include data from three independent experiments.

Upon analyzing the resulting model for the pICoP, it is observed that the model constructs a linear decision boundary (a straight line), which corresponds to the average value of the decision threshold (cut-off line): ${2\;*\;\%CD8}^{+}+{\%CD4}^{+}-{\%CD11b}^{+}{Gr1}^{+}$ = 30.57 ± 0.31. This is visualized as a dashed blue line in the representation of one of the resulting trees (Figure 5A). In the context of a decision-tree model with a depth of 1, this linear decision boundary represents the threshold that divides the data into two distinct regions.

Additionally, a Mann–Whitney U test was used to assess the statistical association between the engineered biomarker and survival-based protection. As shown in Figure 5B, the variable was significantly higher in protected animals, supporting the notion that the strategy of rationally designing biomarkers that integrate both the effector and regulatory arms of the immune system may be valuable for identifying potential vaccine-induced CoPs against *T. cruzi* infection. Taken together, these findings support the initial proposal that combining rational biomarker design with computational analysis and machine-learning optimization can be a valuable strategy for identifying potential CoPs.

### 3.6. Evaluation of the pICoP Decision Logic Under Input Variability

From a data science perspective, it is important to assess whether the generated model, based on a linear combination, is overfitted to the training data and whether small variations in those values could significantly compromise the model’s performance.

To evaluate whether the classification logic defined by the engineered biomarker pICoP remains valid when applied to data that deviates from the original measurements, we conducted an analysis based on simulated input variability. To simulate such variability, we assessed the performance of our analysis pipeline—consisting of model training and evaluation using the pICoP framework and the ROC-AUC as the primary performance metric—on synthetically perturbed versions of the dataset. These synthetic datasets were generated by introducing controlled levels of additive Gaussian noise to the input features, thereby emulating realistic sources of experimental noise and measurement uncertainty. Specifically, stochastic variability was introduced into the original flow cytometry measurements of %*CD*4^+^, %*CD*8^+^, and %*CD*11*b*^+^ *Gr*-1^+^ MDSC-like cells, within predefined ranges (±0–5%, ±5–10%, and ±10–15% of original values), as described in the Methods Section. For each varied dataset, the pICoP was recalculated using the same linear combination of ${2\;*\;\%CD8}^{+}+{\%CD4}^{+}-{\%CD11b}^{+}{Gr1}^{+}$, and a new decision-tree model was trained and evaluated. For each condition, we conducted 100 independent experimental trials, each involving the training of a classification model using pICoP followed by performance evaluation. This process allowed us to examine whether the structure of the biomarker retained its discriminative capacity when exposed to systematically altered input data. As part of the analysis, we quantified the frequency with which the AUC-ROC values exceeded the threshold of 0.7 [38], a value recognized as indicative of acceptable discriminatory performance in biomedical classification tasks.

**Figure 6:**
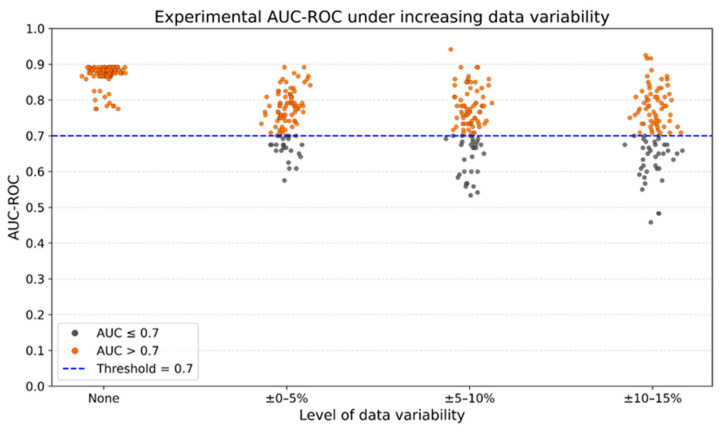
Classification performance of the pICoP decision logic under increasing levels of input variability. Stochastic variability within the ranges of ±0–5%, ±5–10%, and ±10–15% of the original values was applied to the flow cytometry measurements of %*CD*4^+^, %*CD*8^+^, and %*CD*11*b*^+^ *Gr*-1^+^ MDSC-like cells. For each altered dataset, the pICoP was recalculated using the same linear combination (${2\;*\;\%CD8}^{+}+\;{\%CD4}^{+}\;-\;{\%CD11b}^{+}{\;Gr1}^{+}$), and a new decision-tree model was trained and tested. The figure displays individual AUC-ROC values obtained across 100 iterations for each noise level, including the unaltered dataset (No variability). Orange dots represent AUC-ROC values above the established threshold of 0.7—considered acceptable for biomedical data classification—while gray dots correspond to values at or below this threshold.

Figure 6 summarizes the results of these experiments. Each point in the figure represents the average ROC-AUC score for a single trial under a given noise level. As a comparative reference, we also include the performance obtained using different partitioning strategies on the original (non-perturbed) dataset. The results showed that under unaltered conditions, all 100 iterations exceeded this threshold of 0.7. With the introduction of ±0–5% variability, 76 out of 100 models retained acceptable performance; with ±5–10% variability, 70 out of 100; and with ±10–15% variability, 63 out of 100.

These findings indicate that the classification logic embedded in the pICoP formulation remains functional across varying levels of input variability, consistently enabling discrimination between surviving and non-surviving mice by day 25 post-infection in this *T. cruzi* infection model.

## 4. Discussion

Numerous attempts have been made to develop a prophylactic or therapeutic vaccine against *T. cruzi* [7,9,43,44]. However, demonstrating clinical efficacy is challenging, as sterilizing vaccines have not been achievable yet, and assessing the impact of prophylactic vaccination on pathology is difficult, given that only 30–40% of infected patients progress to the symptomatic phase and that pathology typically takes decades to appear [7].

In this context, defining CoPs as substitute endpoints could be highly valuable for accelerating vaccine development, guiding antigen and adjuvant selection, and identifying key immunological elements required for protection. Although antibody levels are the most widely recognized CoPs in vaccine research [16,20] and have been extensively measured in vaccine candidates against *T. cruzi*, no study has yet demonstrated that this biomarker, or any other, could be used as a CoP for any vaccine candidate against this parasite [9]. Thus, novel approaches that take cellular responses into account may be required to improve their identification.

Given that our optimized vaccine candidate double 5FU TSf-ISPA has shown protective capacity associated with an increased effector response in the spleen and lymph nodes, along with decreased MDSC levels [28,30,31], we applied ML in this study to assess potential CoPs using similar cellular biomarkers measured by flow cytometry from the peripheral blood of immunized mice [36], including the percentages of CD4^+^ cells, CD8^+^ cells, and CD11b^+^ Gr-1^+^ MDSC-like cells.

The exploratory analysis and the evaluation of potential CoPs using ML models suggested that individual biomarkers alone are not sufficient to predict infection outcomes in vaccinated mice. To overcome this limitation, a rational combination of biomarkers was developed, based on previous insights, indicating that an interplay between components of the effector and the regulatory response may shape the response elicited by immunization [13,28,30,31,45]. The rationale behind the biomarker engineering performed was as follows: CD4^+^ and CD8^+^ T cells were considered more closely associated with the effector immune response, whereas CD11b^+^ Gr-1^+^ MDSC-like cells were linked to regulatory or suppressive immune functions. This classification aligns with their migratory capacity to the spleen, where their suppressive effects on CD4^+^ lymphocytes and dendritic cells had already been demonstrated [31]. The resulting rationally engineered biomarker (%*CD*8^+^ + %*CD*4^+^− %*CD*11*b*^+^ *Gr*1^+^) demonstrated greater predictive capacity than any of the individual biomarkers. Furthermore, after evaluating the impact of assigning greater weight to CD8^+^ cells, or the other biomarkers, by exhaustive computational analysis plus ML assessment of more than 13,000 combinations, it was observed that all the best alternatives were based on the same strategy of summing up the variables more related to the effector response, giving more weight to the CD8^+^ variable, and subtracting the variable more associated with the regulatory/suppressive response, supporting the original rational research for CoP development. From this analysis, a new potential integrative CoP was selected: ${2\;*\;\%CD8}^{+}+{\%CD4}^{+}-{\%CD11b}^{+}{Gr1}^{+}$ (pICoP), which yielded the highest predictive performance with statistical significance regarding its capacity for classification and its ability to improve the metrics as compared to the initial engineered biomarker ${\%CD8}^{+}+{\%CD4}^{+}-{\%CD11b}^{+}{Gr1}^{+}$ (REB).

This pICoP was based on the concept that such a combination would better reflect the net magnitude of the effector response induced by vaccination, after accounting for the influence of suppressive elements. Strikingly, although the formula was designed through broad generalization and incorporated only a small number of immune components, applying this concept led to a notable improvement in the performance metrics of the decision-tree ML model. Further considerations would be useful to optimize and validate the use of this approach. For example, CD4+ cells measured include all types of CD4+ cells, not just effector cells stimulated by the vaccine. Importantly, CD4+ cells also include Foxp3+ cells with suppressive potential, and CD8^+^ regulatory T cells have also been described [46]. Therefore, considering that CD4^+^ and CD8^+^ cells are related to effector components represents an oversimplification that can be refined. In addition, antigen specificity may be a crucial factor, as certain epitopes could be more relevant than others in shaping the functionality of either the suppressive or effector response. Moreover, although we have reported that CD11b^+^ Gr-1^+^ splenocytes isolated on day 7 post-infection possess the ability to suppress dendritic cell maturation and CD4^+^ T cell proliferation [31], and CD11b^+^ Gr-1^+^ cells that increase in peripheral blood at 48 h post-immunization include cells that home to the spleen (Supplementary Figure S2), not all the cells may exhibit the same behavior as the splenic cells previously assessed [31].

On the other hand, although this study prioritized the analysis of cellular components, incorporating humoral components may further improve the performance of the ML models. Measuring plasma antibodies or cytokines associated with the effector response, such as IL-4, IL-6, IL-17, and IFN-γ, as well as those linked to the suppressive response, including IL-10 and TGF-β, could enhance the identification of the engineered CoPs, lead to further establishment of other CoP, or aid as additional biomarkers that improve the performance of the ML models. Further analyses are ongoing to continue advancing ML applications in the design of the double 5FU TSf-ISPA vaccine.

Regardless of potential future improvements, the pICoP developed led to a notable improvement in all assessed metrics, including accuracy, AUC-ROC, F1-score, precision (Supplementary Table S1), and recall (Supplementary Table S1), in both the training and test sets of the decision-tree model used. The values of the AUC-ROC metric obtained when analyzing biomedical data have been classified as low discriminative from 0.5 to 0.7, acceptable from 0.7 to 0.8, excellent from 0.8 to 0.9, and outstanding from 0.9 to 1 [38]. According to this scale, the use of the pICoP improved the performance of the decision-tree model to 0.87 AUC-ROC, which corresponds to an excellent level of prediction [38]. This level of performance is highly promising given that no mechanistic or statistical CoP has been identified yet for a *T. cruzi* vaccine.

Additionally, this level of classification is comparable to the metrics reported in other studies using antibody-based CoPs [47,48,49] and traditional statistical approaches. Thus, these results provide evidence that rational approaches—integrating cellular biomarkers from the immune system, rather than focusing solely on effector responses—can aid in identifying potential CoPs for a *T. cruzi* vaccine through ML.

ML also opens the possibility to explore new engineered biomarkers through a wide combination of existing variables. In this case, prior knowledge and rational biomarker engineering, combined with computational variable analysis and ML techniques, were instrumental in identifying the most promising CoP. Although the present study involves a relatively small number of biomarkers, the observed results already demonstrate the value of this approach. As the dimensionality increases in future studies, including other biomarkers such as cytokine levels, antibody titers, or additional immune cells, ML-driven strategies are likely to become even more valuable for CoP discovery, offering scalable and systematic solutions to navigate the growing combinatorial complexity inherent to vaccine research.

Most studies aiming to identify CoPs have employed logistic regression, fitting models to the entire dataset. This approach requires relatively large sample sizes and relies on the assumption that the sample is representative of the population. In contrast, ML models—optimized through training on data and testing on separate, unseen datasets—do not depend on this assumption. This strategy not only relaxes distributional requirements but also improves generalizability and the potential applicability of identified CoPs in future studies. Furthermore, logistic regression models treat biomarkers in an additive manner, limiting their capacity to capture complex interactions. ML algorithms such as decision trees, random forests, neural networks, and others can simultaneously analyze multiple biomarkers, better accounting not only for linear but also for non-linear relationships and laying the groundwork for improved CoP identification through the inclusion of additional biomarkers that may or may not be CoPs themselves. The development and use of these models could be a valuable tool in the near future for predicting the immunological outcome of vaccines when univariate CoPs prove insufficient and multivariate analysis may be required.

To assess the consistency of the decision logic defined by the pICoP when applied to datasets different from those originally used, we simulated input variability by introducing controlled stochastic changes to the original flow cytometry values of %*CD*4^+^, %*CD*8^+^, and %*CD*11*b*^+^ *Gr*-1^+^ MDSC-like cells. The engineered biomarker was then recalculated using the linear combination (${2\;*\;\%CD8}^{+}+{\%CD4}^{+}-{\%CD11b}^{+}{Gr1}^{+}$), and a new decision-tree model was trained and tested on each modified dataset. This strategy allowed us to evaluate whether the structural formulation of the pICoP retains its discriminative capacity under plausible variations in input data. Despite increasing levels of variability, the classification performance remained consistently acceptable in most cases, with a substantial proportion of models exceeding the AUC-ROC threshold of 0.7 [38]. These results support the functional stability of the pICoP’s underlying logic, reinforcing its potential utility as a reliable CoP of protection in this *T. cruzi* infection model.

We have already shown that the double 5FU TSf-ISPA vaccine shows protective capacity in multiple preclinical models, including the use of Tulahuen (DTU VI) and Dm28c (DTU I) *T. cruzi* strains, using BALB/c and C57BL/6 immune background mice, and different doses and routes of infection [31]. Thus, analyzing the predictive capacity of the pICoP in the different preclinical models would serve to further support the robustness of the identified pICoP.

Recently, it has been proposed that polyfunctional T cells could serve as CoPs for *T. cruzi* vaccines [50]. In the same line, it has been reported that protection against pox-virus, Leishmania major malaria, and tuberculosis may also be T-cell-mediated, with various functions such as IFN-γ, IL-2, TNF-α, and granzyme B being potentially involved [51,52,53,54,55]. While these hypotheses may be valid, measuring all those markers and immune populations in mouse preclinical models could not be straightforward due to low frequency in blood, the requirement for intracellular staining, and the substantial blood volume necessary for such analyses in preclinical models. Herein, we describe a simple protocol for measuring multiple immune cell populations that are present at high percentages in peripheral blood, providing a straightforward and reliable methodology for identifying potential CoPs. Although four-color flow cytometry was used in this study, the employment of multiparametric analysis may significantly enhance this approach. Additionally, this study used mortality as the clinical endpoint, as it represents a clear and reliable measure of vaccine-induced protection in murine models. However, future studies are expected to incorporate additional parameters such as histopathology and parasitemia, in order to begin evaluating variables that are also used to assess disease progression in human infection.

We have recently reviewed that MDSC cell increases are not exclusive to our model. In contrast, several adjuvants and routes of immunization have been shown to induce MDSCs, limiting the efficacy of other vaccines [56]. According to this, depleting MDSCs may become a valuable strategy to improve vaccine efficacy [30,31,56]. Moreover, targeting regulatory T cells (Tregs) has emerged as a promising strategy to enhance vaccine-induced protection [57,58,59]. Several studies using different vaccination models have demonstrated that transient Treg depletion can boost antigen-specific immune responses in vaccines against viruses [60,61], bacteria [62], fungi [63,64,65,66], and parasites [67]. In these models, Treg depletion has been associated with enhanced effector immune mechanisms, particularly involving Th1, Th17, and CD8^+^ T cell responses, which are critical for effective pathogen clearance. These findings support the notion that modulating the immunosuppressive activity of Tregs may be a valuable approach to improve the efficacy of anti-infectious vaccines. In this context, greater attention should be given to the integration of regulatory circuits, primarily governed by Tregs and MDSCs, which can critically shape vaccine-induced immunity. A deeper understanding of how regulatory populations shape the magnitude, quality, and durability of protective immune responses is critical for the rational design of next-generation vaccines and should be integrated into the analysis of CoPs [56,57,58,59,60].

To our knowledge, this is the first report describing a novel strategy that combines rational biomarker engineering, computational analysis, and machine learning for the analysis of cellular immune biomarkers in order to facilitate the identification of CoPs. This approach may offer a rapid, ethically justifiable, and practical tool for vaccine development against complex pathogens for which no vaccines currently exist, providing insights into protection-associated biomarkers and ultimately guiding and accelerating vaccine research to improve human health.

## 5. Conclusions

This study provides a working methodology that supports the use of rational cellular biomarker engineering, combined with machine learning, as a valuable tool for identifying potential vaccine CoPs against *T. cruzi*. The proposed pICoP, which integrates effector and regulatory immune cellular markers, significantly improved the predictive performance of the protective capacity of the vaccine candidate double 5fU TSF-ISPA, compared with individual biomarkers. These results highlight the importance of considering both effector and suppressive immune components in vaccine research. Several plausible avenues exist to refine the pICoP, including the incorporation of new biomarkers such as antibody titers and plasma cytokine levels. In addition, evaluating the pICoP in other preclinical models will allow assessment of its robustness. Finally, it is worth noting that the proposed framework could also be applied to other infectious models where classical antibody-based CoPs have not yet been sufficiently effective. In this way, integrating immunological knowledge with machine learning could accelerate the rational design of vaccines against complex pathogens.

## Figures and Tables

**Table 1:** Average performance evaluation of individual and rationally engineered biomarkers as potential correlates of protection (CoPs) using decision-tree models.

Potential CoP	F1-Score of Alive Mice	Accuracy	AUC-ROC
${\%CD8}^{+}$	0.47 [95% CI: 0.46–0.49]	0.47 [95% CI: 0.46–0.49]	0.58 [95% CI: 0.57–0.59]
${\%CD4}^{+}$	0.65 [95% CI: 0.64–0.66]	0.61 [95% CI: 0.61–0.62]	0.68 [95% CI: 0.67–0.69]
${\%CD11b}^{+}{Gr1}^{+}$	0.50 [95% CI: 0.49–0.51]	0.50 [95% CI: 0.48–0.51]	0.53 [95% CI: 0.52–0.55]
${\%CD8}^{+}+{\%CD4}^{+}-{\%CD11b}^{+}{Gr1}^{+}$ (REB)	0.61 [95% CI: 0.59–0.63]	0.72 [95% CI: 0.71–0.73]	0.70 [95% CI: 0.69–0.71]

**Table 2:** Average performance evaluation of both engineered biomarkers using decision-tree models.

Potential CoP	F1-Score of Alive Mice	Accuracy	AUC-ROC
${\%CD8}^{+}+{\%CD4}^{+}-{\%CD11b}^{+}{Gr1}^{+}$ (REB)	0.61 [95% CI: 0.59–0.63]	0.72 [95% CI: 0.71–0.73]	0.70 [95% CI: 0.69–0.71]
${2\;*\;\%CD8}^{+}+{\%CD4}^{+}-{\%CD11b}^{+}{Gr1}^{+}$ (pICoP)	0.83 [95% CI: 0.82–0.84]	0.86 [95% CI: 0.86–0.87]	0.87 [95% CI: 0.86–0.87]

## Data Availability

All data associated with this study are available in Supplementary Material SI (dataset.csv) and SII (pICoP_search.csv) at https://doi.org/10.5281/zenodo.16281869. Source code for the experiments presented in this work is available at https://github.com/JuanCruzGamba/PhD_repository/tree/main/publication_projects/integrative_CoP_with_ML (accessed on 25 August 2025).

## Supplementary Materials

The following supporting information can be downloaded at: https://www.mdpi.com/article/10.3390/vaccines13090915/s1.

## Abbreviations

The following abbreviations are used in this manuscript:

*T. cruzi*	*Trypanosoma cruzi*
CoP	Correlate of Protection
mCoP	Mechanistic Correlate of Protection
nCoP	Non-Mechanistic Correlate of Protection
ML	Machine Learning
TSf	Trans-Sialidase Fragment
5FU	5-Fluorouracil
MDSC	Myeloid-Derived Suppressor Cell
CD4^+^	Cluster of Differentiation 4 Positive
CD8^+^	Cluster of Differentiation 8 Positive
CD11b^+^	Cluster of Differentiation 11b Positive
Gr-1^+^	Granulocyte marker 1 Positive
ISPA	Cage-Like Particle Adjuvant
PBS	Phosphate-Buffered Saline
CFSE	5,6-Carboxyfluorescein Diacetate Succinimidyl Ester
REB	Rational Engineered Biomarker
pICoP	Potential Integrative Correlate of Protection
AUC-ROC	Area Under the Receiver Operating Characteristic Curve
Treg	Regulatory T Cell
DC	Dendritic Cell
