# Supplementary material for: Integrating Cellular Immune Biomarkers with Machine Learning to Identify Potential Correlates of Protection for a Trypanosoma cruzi Vaccine
Source: Vaccines (Basel). 2025 Aug 28;13(9):915. doi: 10.3390/vaccines13090915 (PMC12474346; doi:10.3390/vaccines13090915)
Supplement: Supplementary file 1 [file vaccines-13-00915-s001.zip › Supplementary table I.pdf]

Classification performance metrics for pICoP with 95% confidence intervals

Training set evaluation metrics

	Precision	Recall	F1-Score
Class 0 (Death mice)	0.94 [95% CI: 0.94 – 0.94]	0.84 [95% CI: 0.84 – 0.84]	0.89 [95% CI: 0.89 – 0.89]
Class 1 (Alive mice)	0.79 [95% CI: 0.79 – 0.79]	0.92 [95% CI: 0.92 – 0.92]	0.85 [95% CI: 0.85 – 0.85]
Average Accuracy		0.87 [95% CI: 0.87 – 0.87]	
Average AUC-ROC		0.88 [95% CI: 0.88 – 0.88]	

Test set evaluation metrics

	Precision	Recall	F1-Score
Class 0 (Death mice)	0.94 [95% CI: 0.93 – 0.94]	0.84 [95% CI: 0.83 – 0.84]	0.87 [95% CI: 0.87 – 0.88]
Class 1 (Alive mice)	0.80 [95% CI: 0.79 – 0.81]	0.90 [95% CI: 0.89 – 0.91]	0.83 [95% CI: 0.82 – 0.84]
Average Accuracy		0.86 [95% CI: 0.86 – 0.87]	
Average AUC-ROC		0.87 [95% CI: 0.86 – 0.87]	
