# Supplementary figures and images for: Integrating Cellular Immune Biomarkers with Machine Learning to Identify Potential Correlates of Protection for a Trypanosoma cruzi Vaccine
Source: Vaccines (Basel). 2025 Aug 28;13(9):915. doi: 10.3390/vaccines13090915 (PMC12474346; doi:10.3390/vaccines13090915)

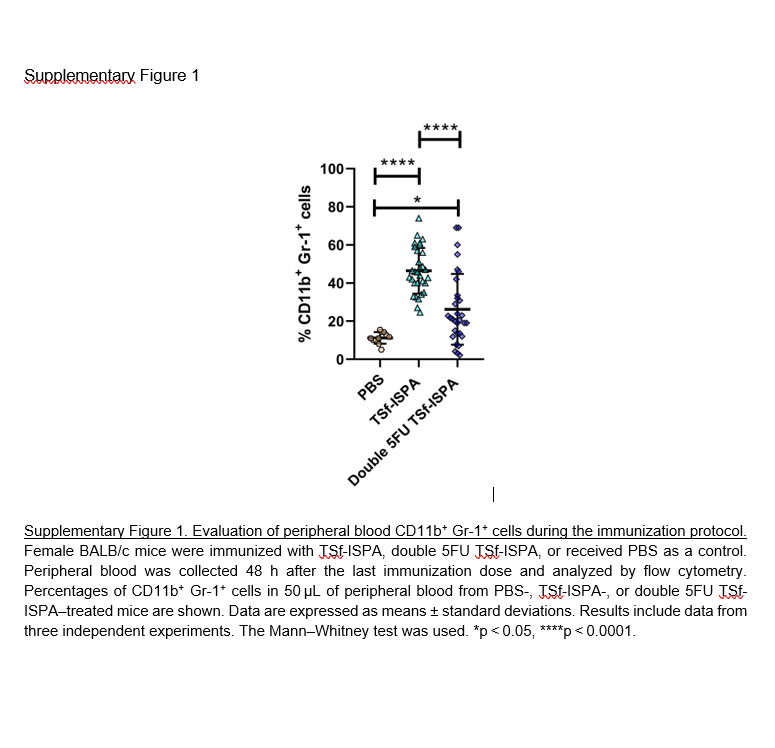

Supplement: Supplementary file 1 [file vaccines-13-00915-s001.zip › Supplementary Figure 1.tif]

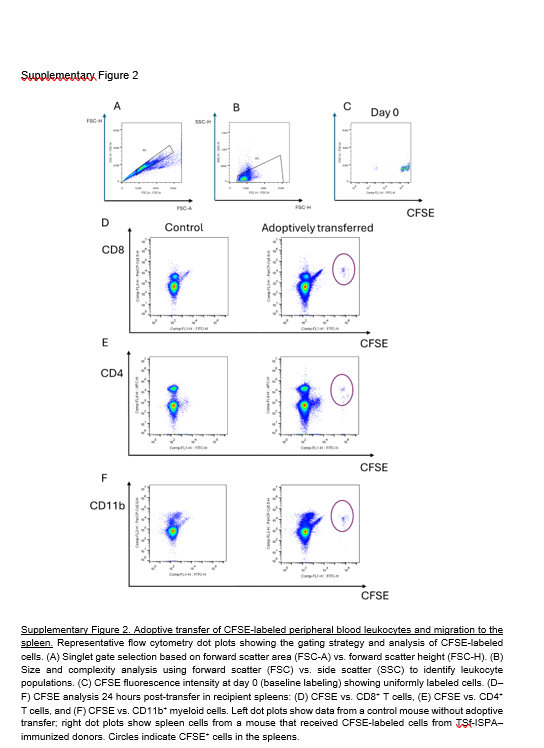

Supplement: Supplementary file 1 [file vaccines-13-00915-s001.zip › Supplementary Figure 2.tif]
